# Dye Sensitizers for Photodynamic Therapy

**DOI:** 10.3390/ma6030817

**Published:** 2013-03-06

**Authors:** Alexandra B. Ormond, Harold S. Freeman

**Affiliations:** Fiber and Polymer Science Program, North Carolina State University, Raleigh, NC 27695-8301, USA; E-Mail: hfreeman@ncsu.edu

**Keywords:** photodynamic therapy, photosensitizers, porphyrins, clinical trials, target organs

## Abstract

Photofrin^®^ was first approved in the 1990s as a sensitizer for use in treating cancer via photodynamic therapy (PDT). Since then a wide variety of dye sensitizers have been developed and a few have been approved for PDT treatment of skin and organ cancers and skin diseases such as *acne vulgaris*. Porphyrinoid derivatives and precursors have been the most successful in producing requisite singlet oxygen, with Photofrin^®^ still remaining the most efficient sensitizer (quantum yield = 0.89) and having broad food and drug administration (FDA) approval for treatment of multiple cancer types. Other porphyrinoid compounds that have received approval from US FDA and regulatory authorities in other countries include benzoporphyrin derivative monoacid ring A (BPD-MA), *meta*-tetra(hydroxyphenyl)chlorin (*m*-THPC), *N*-aspartyl chlorin e6 (NPe6), and precursors to endogenous protoporphyrin IX (PpIX): 1,5-aminolevulinic acid (ALA), methyl aminolevulinate (MAL), hexaminolevulinate (HAL). Although no non-porphyrin sensitizer has been approved for PDT applications, a small number of anthraquinone, phenothiazine, xanthene, cyanine, and curcuminoid sensitizers are under consideration and some are being evaluated in clinical trials. This review focuses on the nature of PDT, dye sensitizers that have been approved for use in PDT, and compounds that have entered or completed clinical trials as PDT sensitizers.

## 1. Introduction

In the medical arena, the treatment of skin diseases with the aid of light has been performed since 1400 BC [1], and this technology is now known as phototherapy. Phototherapy employs either UV or visible light, with or without a photosensitizer—A molecule capable of absorbing light energy and transferring that energy to adjacent molecules. When a photosensitizer is not used, phototherapy is mainly employed in dermatology to treat vitamin D deficiency, neonatal jaundice, psoriasis, eczema, vitiligo, polymorphous light eruption, cutaneous T-cell lymphoma, lichen planus, and even to ease the symptoms of Parkinson’s disease [2,3,4].

**Figure 1:**
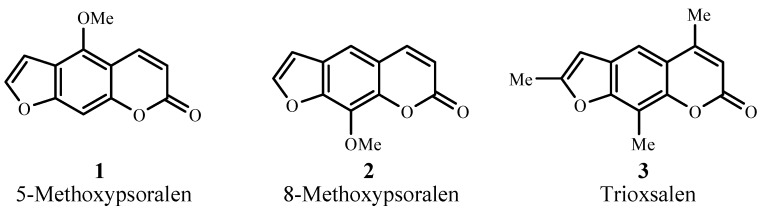
Examples of psoralen photosensitizers.

Photochemotherapy, on the other hand, utilizes a photosensitizer, usually of the psoralen series (**1–3**; Figure 1), in tandem with UVA (300–400 nm) radiation [5]. Treatments involve psoriasis, atopic dermatitis, seborrheic dermatitis, eczema, alopecia areata, chronic cutaneous graft-versus-host disease, HIV-associated dermatoses, histiocytosis, lichen planus, mycosis fungoids, polymorphous light eruption, pityriasis lichenoides, lymphamatoid papulosis, prurigo, palmar and plantar pustulosis, and vitiligo [6].

**Figure 2:**
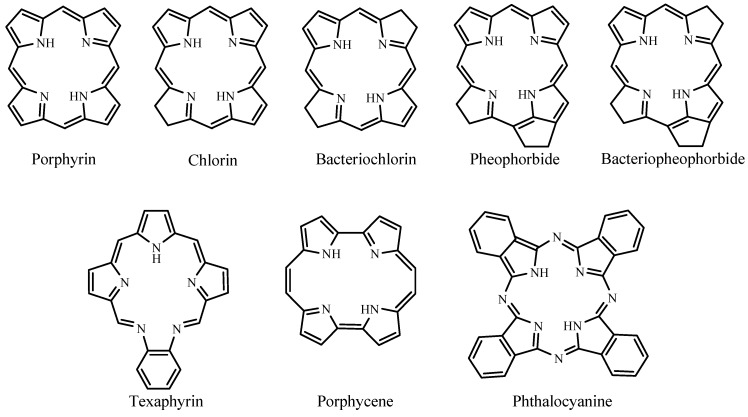
Basic structures of porphyrinoid photosensitizers.

Photodynamic therapy (PDT) is a type of photochemotherapy and requires the presence of light, a photosensitizer, and molecular oxygen for treatments [7]. The combination of photosensitizer and light as therapeutic agents was first introduced in the early 1900s [8] but it was not until the 1990s that the food and drug administration (FDA) approved PDT using a pure form of Photofrin^®^. Suitable dye sensitizers for PDT are mainly porphyrinoid compounds, including chlorins, bacteriochlorins, phthalocyanines, and related structures [9]. These compounds have extended conjugation and absorb light in the visible region, which makes them colored compounds or dyes. This review covers porphyrinoid and non-porphyrin dye photosensitizers. The porphyrinoid photosensitizers reviewed are porphyrins, chlorins, pheophorbides, bacteriopheophorbides, texaphyrins, and phthalocyanines. The non-porphyrins are anthraquinones, phenothiazines, xanthenes, cyanines, and curcuminoids. Figure 2 shows the basic structures of porphyrinoid compounds.

### 1.1. Photodynamic Action and Mechanisms

**Figure 3:**
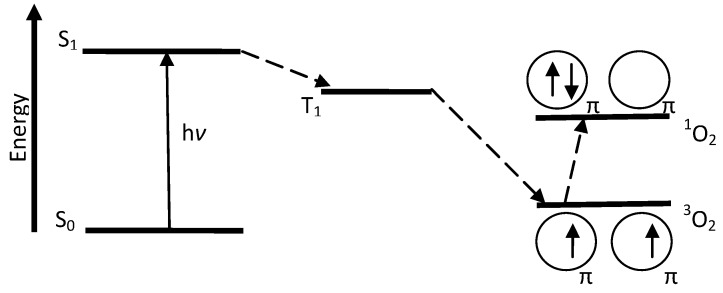
Modified Jablonski diagram showing Type II sensitization process.

PDT involves the use of light exposures to transform a sensitizer from the ground state (S_0_) to the first excited state (S_1_). The sensitizer must be sufficiently stable in the excited state to undergo intersystem crossing to the triplet excited state (T_1_), a longer-lived state. At this stage, two reaction processes involving molecular oxygen can take place. In the first process, Type I, hydrogen abstraction or electron transfer between an excited sensitizer and an adjacent sensitizer molecule occurs, with ion radical formation. The resultant radical can react with ground state oxygen (^3^O_2_) to produce reactive oxygen species (ROS) such as superoxide anion (O_2_^−^^•^), hydrogen peroxide (H_2_O_2_), and hydroxyl radical (OH^•^) [9]. In the second process, Type II, energy from T_1_ is transferred directly to ^3^O_2_, exciting it to singlet oxygen (^1^O_2_) as illustrated in Figure 3. Energy transfer to ^3^O_2_ can occur only if a sensitizer is in the same triplet state multiplicity, or occupies T_1_, as ground state oxygen.

Table 1 lists the series of reactions that occur during PDT. PS is the photosensitizer, ^1^PS is PS in ground state, ^1^PS* and ^3^PS* are PS in singlet excited and triplet excited states, respectively, and D is an electron donor molecule, e.g., NADH, cysteine, *etc*. [9]. The reaction between ^3^PS* and ^1^PS leads to PS anion and cation radicals, PS^−•^ and PS^+•^, respectively. D can react with ^3^PS* to produce more PS^−•^ and oxidized donor (D^+^). The superoxide anion, O_2_^−•^, is shown to form via two routes: (1) PS^−•^ electron exchange with oxygen and (2) electron transfer of ^3^PS* with oxygen. O_2_^−•^ formation from ^3^PS*, however, competes with the production of singlet oxygen (type II). Also, two superoxide anion molecules can combine with protons to produce hydrogen peroxide. The subsequent steps include reduction of Fe^3+^ by O_2_^−•^, and Fe^2+^ reaction with hydrogen peroxide to form a hydroxyl radical. This species can interfere with the biological functions of nucleic acids, fatty acids, and certain amino acids [9].

Type II process involves only a limited number of molecules because the reacting species must have triplet state multiplicity [8]. Since ground state oxygen (^3^O_2_) is already in its triplet state, the reaction between triplet excited state of a photosensitizer and ^3^O_2_ is possible. Type I and Type II processes occur at the same time; however, Type II is the dominant process in PDT and it is catalytic.

**Table 1:** Reactions occurring during photodynamic action [9].

Excitation		^1^PS + hν → ^1^PS* → ^3^PS*		
**Photoprocess**	**Reaction**			**Product**
Type I	^3^PS* + ^1^PS		→	PS^−•^ + PS^+•^
	^3^PS* + D		→	PS^−•^ + D^+^
	PS^−•^ + O_2_		→	^1^PS + O_2_^−•^
	^3^PS* + O_2_		→	PS^+•^ + O_2_^−•^
	2O_2_^−•^ + 2H^+^		→	O_2_ + H_2_O_2_
	Fe^3+^ + O_2_^−•^		→	Fe^2+^ + O_2_
	Fe^2+^ + H_2_O_2 _		→	O_2_ + OH^−^ + OH^•^
Type II	^3^PS* + ^3^O_2_		→	^1^PS + ^1^O_2_

#### 1.1.1. Photodynamic Action in the Body

**Figure 4:**
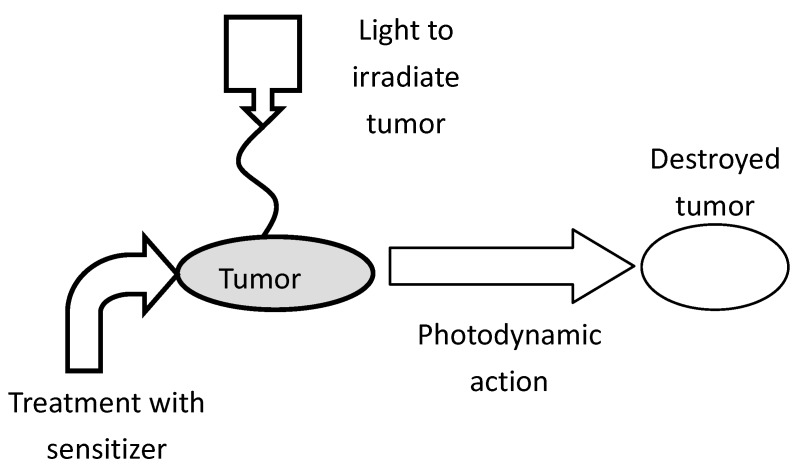
Schematic representation of photodynamic therapy (PDT) treatment of a malignant tumor [7].

During PDT, a sensitizer can be administered intravenously, intraperitoneally, or topically, and it selectively localizes in a tumor due to physiological differences in the tumor and healthy tissue [10,11]. Localization into cancer cells and achieving a maximum tumor-to-normal cell concentration ratio can take 3 to 96 h, depending on the photosensitizer and the tumor. Following localization, fluorescence from the sensitizer can be used to diagnose and detect the tumor. Irradiation at a wavelength specific to the photosensitizer produces singlet oxygen, which reacts with and destroys the tumor (cf. Figure 4). Cell destruction can occur in several ways, one of which is damage to the vasculature by erythema or edema, another is direct cell destruction by apoptosis or necrosis [12,13]. Chances of skin photosensitivity are high, even though the dye has greater affinity for tumor tissue. This effect requires patients to limit sunlight exposure to eyes and skin up to thirty days or longer following treatment [14], depending on the sensitizer. 

### 1.2. Light and Oxygen in PDT

**Figure 5:**
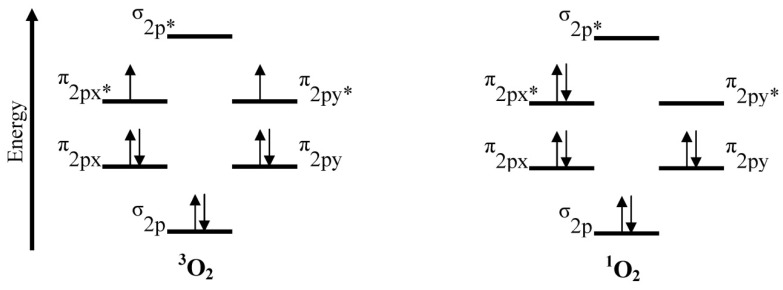
Triplet (^3^O_2_) and singlet (^1^O_2_) states of oxygen.

Light can be delivered via an argon or copper pumped dye laser coupled to an optical fiber, a double laser consisting of KTP (potassium titanyl phosphate)/YAG (yttrium aluminum garnet) medium, LED (light emitting diode), or a solid state laser [13]. For PDT using Photofrin^®^ as the photosensitizer, an argon pumped dye laser coupled to an optical fiber is used and it operates at 630 nm. At this wavelength, light penetrates only 2 to 3 mm into the tissue. The ideal photosensitizer is activated by light absorption at 700–800 nm, and provides light penetration of 5 to 6 mm depths [12]. At low wavelengths, scattering and absorption of light by human tissue is high, and at wavelengths far into the red or near infrared regions, negative effects are also possible. One negative effect is photobleaching [2]. Photobleaching causes dye sensitizer modification with or without chromogen destruction, as well as loss of fluorescence [15]. In the case of Photofrin^®^, a decrease in oxidation potential and photostability occurs [2]. Another consequence of light absorption at higher wavelength is inefficient energy transfer from T_1_ of the photosensitizer during singlet oxygen generation [15]. With appropriate energy transfer, ground state oxygen is converted to singlet oxygen (cf. Figure 5). This transition requires 94 kJ mol^−1^ (22.5 kcal mol^−1^) or 1270 nm; thus, triplet states of photosensitizers extending beyond this region will not have enough energy to produce singlet oxygen.

The lifetime of singlet oxygen is very short due to its reactivity. In H_2_O, the lifetime is 3.5 μs, in D_2_O it is 68 μs [16], in organic solvents its lifetime is 10–100 μs [8], and in lipids it is 50–100 μs [17]. The lifetime decreases dramatically to 0.2 μs inside cells, due to high reactivity with biological substances [9]. Rapid reactivity and a short lifetime limit the singlet oxygen distribution in cells. Thus, PDT treatments are localized at the point of ^1^O_2_ generation and are only about 10 nm in diameter (thickness of a cell membrane) [8,18].

### 1.3. Photosensitizer Distribution in Tissues

Inside the body, photosensitizers probably interact with tumors via low-density lipoprotein (LDL) receptors [19]. Cancer cells have elevated levels of LDL receptors; thus, endocytosis of LDL-photosensitizer complex is preferred by malignant cells [20]. Additionally, a high fraction of tumor-associated macrophages is found in these cells, with photosensitizer levels also high in these areas [21,22]. Further selective uptake of dye photosensitizer by tumor cells is possibly due to lower intracellular pH, leaky microvasculature and poor lymphatic drainage by tumors, and large amounts of collagen [17].

Photosensitizer solubility is another important factor in its distribution and location inside tumor cells. Hydrophobic compounds and their aggregates bind to LDL while hydrophilic species bind to albumin and globulins [22,23]. Accumulation of photosensitizers in the cell organelles also depends on the charge of the sensitizer. Cationic compounds, e.g., iminium salts, collect in mitochondria, while anionic species, e.g., sulfonated and carboxylated compounds, are found in lysosomes [22,23]. Dye sensitizers with one or two anionic charges localize in the perinuclear region, vesicles of the cell, and lysosomes, providing multiple sites of photosensitizer accumulation [24,25]. While water solubility is important for bioavailability of the sensitizer, lipophiliciy is important for diffusion through lipid barriers and localization in endocellular cites [26].

## 2. Photosensitizer Types

**Figure 6:**
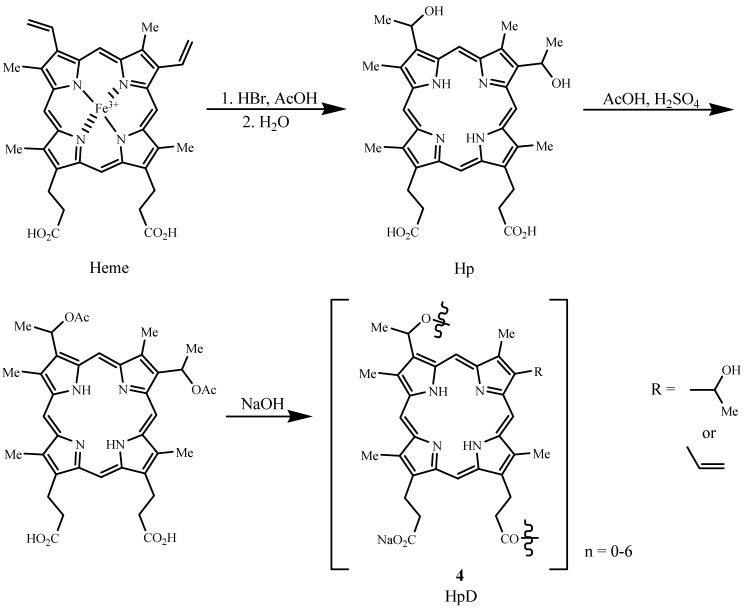
Synthesis of hematoporphyrin derivative (HpD) from heme.

Photofrin^®^ (porfimer sodium), the first FDA approved PDT sensitizer, belongs to the porphyrin family and is a hematoporphyrin derivative (HpD). Hematoporphyrin (Hp) was produced by Scherer in 1841 by removing iron from blood (Heme) and then treatment with water [11]. HpD (**4**) was developed by treating Hp with AcOH/H_2_SO_4_ to give a mixture of monomers, dimers, and oligomers, linked by ether, ester, and carbon-carbon bonds [27]. The types of steps associated with its synthesis are illustrated in Figure 6 [9,28]. Removal of monomers from HpD by heating the reaction mixture in the last step of the synthesis until hydrolysis is complete led to Photofrin^®^, a product consisting of ether-linked dimers and trimers [11,29]. 

### 2.1. Photosensitizer Properties

The ideal PDT photosensitizer has the following characteristics [30]:

(a) available in pure form, of known chemical composition;
(b) synthesizable from available precursors and easily reproduced;
(c) high singlet oxygen quantum yield (Φ_Δ_);
(d) strong absorption in the red region of the visible spectrum (680–800 nm) with a high extinction coefficient (ε_max_), e.g., 50,000–100,000 M^−1^ cm^−1^;
(e) effective accumulation in tumor tissue and possession of low dark toxicity for both photosensitizer and its metabolites;
(f) stable and soluble in the body’s tissue fluids, and easy delivery to the body via injection or other methods;
(g) excreted from the body upon completion of treatment.

### 2.2. First Generation Photosensitizers

Photofrin^®^ and HpD are known as first generation photosensitizers mainly because they exist as complex mixtures of monomeric, dimeric, and oligomeric structures, and the intensity of light absorption at the maximum wavelength (ε_max_) of Photofrin^®^ is low (ε_max_ at 630 nm~3000 M^−1^ cm^−1^). This low ε_max_ means that Photofrin^®^ absorbs light weakly at 630 nm. The higher the ε_max_ value the greater the potential photodynamic effect. Also, at 630 nm, the effective tissue penetration of light is small, 2–3 mm, limiting treatment to surface tumors. Its long-term skin phototoxicity lasts six to ten weeks, meaning sunlight and strong artificial light exposure must be avoided during this period. Although Photofrin^®^ has its weaknesses, it gives a high singlet oxygen quantum yield, Φ_Δ_ = 0.89, which indicates efficient generation of ^1^O_2_ per photon absorbed. Photofrin^®^ is also safe and was approved in 1993 by Canada for treatment of bladder cancer and by the US FDA for treating esophageal cancer in 1995, lung cancer in 1998, and Barrett’s esophagus in 2003 [31]. Photofrin^®^ treatment extends to head, neck, abdominal, thoracic, brain, intestinal, skin, breast, and cervical cancer [30].

Other types of hematoporphyrin derivatives are Photogem^®^ and Photosan-3^®^. Photogem^®^ consists of monomers, dimers, and oligomers [32] and has been approved for use in clinical applications in Russia and Brazil [33]. Photosan-3^®^ has been approved for clinical use in the EU [34].

### 2.3. Second Generation Photosensitizers

The properties of unfavorable skin phototoxicity, low absorption in the red region of the visible spectrum, as well as complex mixtures arising from the method of synthesis were targeted for improvement with second generation photosensitizers. 

#### 2.3.1. Porphyrins

**Figure 7:**
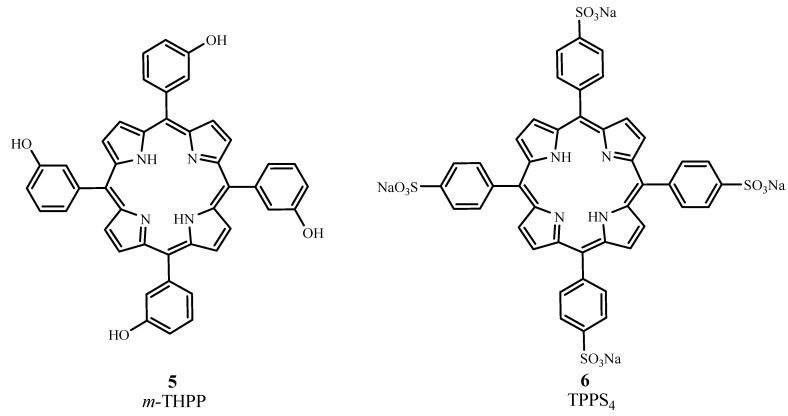
Molecular structures of some second generation porphyrins.

From the porphine family, *meta*-tetra(hydroxyphenyl)porphyrin (*m*-THPP, **5**), the *meta* isomer of 5,10,15,20-tetra(hydroxyphenyl)porphyrin, and 5,10,15,20-tetrakis(4-sulfanatophenyl)-21H,23H-porphyrin (TPPS_4_, **6**) are second generation PDT sensitizers (cf. Figure 7). *m*-THPP, however, caused skin phototoxicity, but not as severely as the *ortho* isomer. *m*-THPP was 25 to 30 times as potent as HpD in tumor photonecrosis when irradiated at 648 nm [2]. TPPS_4_ exhibited lower photochemical efficiency than *meso*-substituted porphyrins containing fewer sulfonate groups [27,30]. The irradiation of TPPS_4_ at 645 nm with an argon-pumped dye laser showed it to be a potential candidate for treating basal cell carcinoma [35].

**Figure 8:**
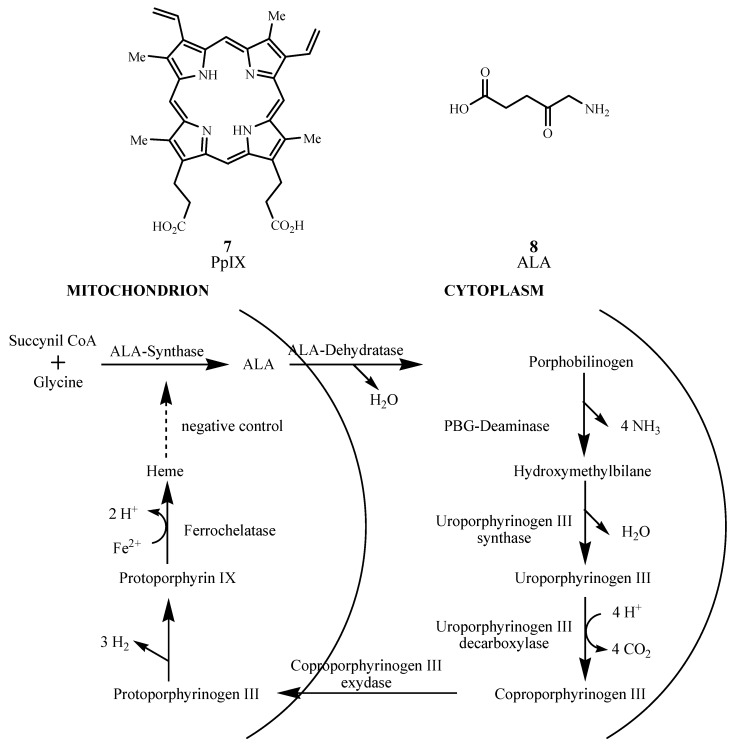
Pathway for heme biosynthesis.

Endogenous Protoporphyrin IX (PpIX, **7**) induced by exogenous 1,5-aminolevulinic acid (ALA or Levulan Kerasticks^®^, **8**) was US FDA approved for non-oncological PDT treatment of actinic keratosis in 1999 [36]. Application of ALA prodrug to skin enzymatically transforms it to PpIX photosensitizer via the heme pathway shown in Figure 8. The final step in heme formation by enzyme ferrochelatase is a rate-limiting step, and excess ALA accumulates PpIX in the mitochondria before it slowly transforms into heme [37]. While the PpIX absorption maximum is low (630 to 635 nm), it metabolizes within 48 hours, reducing skin sensitization [38]. Its potential PDT applications extend to Bowen’s disease, basal cell carcinoma, and other diseases; and ALA can be used to detect tumors in bladder, skin, lung, and gastrointestinal tract [39].

**Figure 9:**
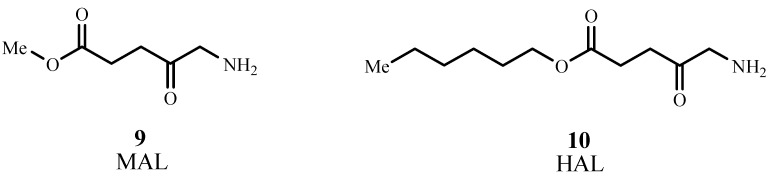
Molecular structures of methyl aminolevulinate (MAL) and Hexaminolevulinate (HAL).

The methyl ester of ALA, methyl aminolevulinate (MAL, Metvix^®^, or Metvixia^®^, **9**; Figure 9), was approved by the US FDA in 2004 for treatment of actinic keratosis [40]. Under the trade name Metvixia^®^, MAL is also used as a topical treatment and has an advantage over Levulan^®^ due to the nature of the irradiation source. Blu-U^®^ light was approved for use with Levulan^®^ as the most efficient source emitting at 400 nm, while Aktilite^®^ was approved for Metvixia^®^ which emits at 630 nm and provides deeper tissue penetration [41]. MAL is the active component in Visonac^®^ and is being studied for acne vulgaris in Phase II trials (NCT01347879) in the US [42].

Hexaminolevulinate, the *n*-hexyl ester of ALA, (HAL, Hexvix^®^, Cysview^®^, **10**) was approved in 2010 by the US FDA in the diagnosis of bladder cancer [43]. HAL is converted to PpIX 50–100 times more efficiently than ALA [44]. Phase II trials are underway for treatment of cervical intraepithelial neoplasia (NCT01256424) [45], and Phase II/III trials are ongoing for genital erosive lichen planus (NCT01282515) [46].

#### 2.3.2. Chlorins

**Figure 10:**
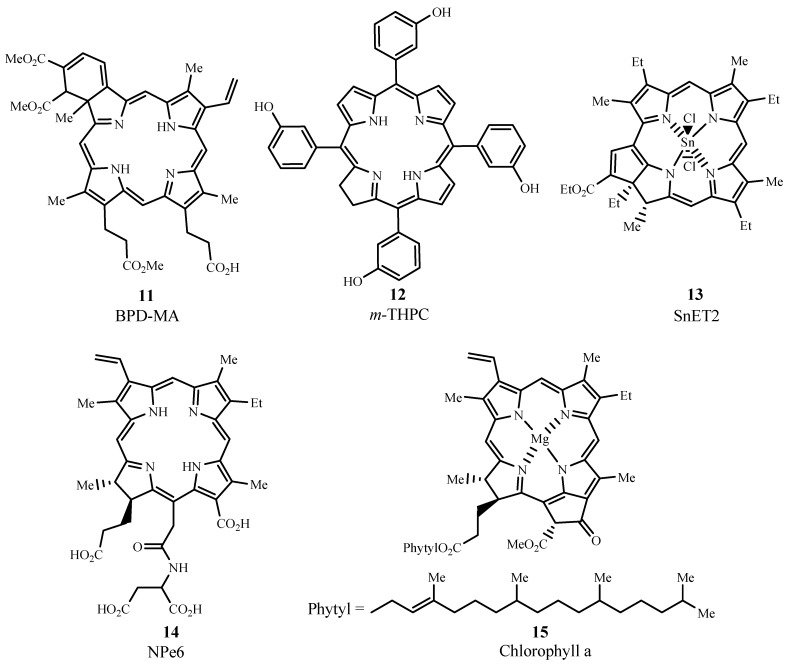
Examples of chlorins evaluated for PDT use.

Several photosensitizers evaluated for PDT efficacy are from the chlorin family (cf. Figure 10) and include benzoporphyrin derivative monoacid ring A (BPD-MA, Verteporfin, Visudyne^®^, **11**), *meta*-tetra(hydroxyphenyl)chlorin (*m*-THPC, Foscan^®^, **12**), tin ethyl etiopurpurin (SnET2, Rostaporfin, Purlytin™, **13**), and *N*-aspartyl chlorin e6 (NPe6, Talaporfin, Ls11, **14**) which is derived from chlorophyll a (**15**). When compared to porphyrins, the structure of chlorins differs by two extra hydrogens in one pyrrole ring. This structural change leads to a bathochromic shift in the absorption band (640 to 700 nm) and gives ε_max_ ~ 40,000 M^−1^ cm^−1^. 

BPD-MA is activated by light at 689 nm and has a lower time interval of skin phototoxicity than Photofrin^®^, due to rapid plasma and tissue pharmacokinetics which enables faster excretion of the drug from the body [47]. In 1999, US FDA approved the use of BPD-MA as Visudyne^®^ for age-related macular degeneration in ophthalmology [48]. Additionally, a 24-month study of Verteporfin treatment showed improvement in patients with non-melanoma skin cancer [49].

**Figure 11:**
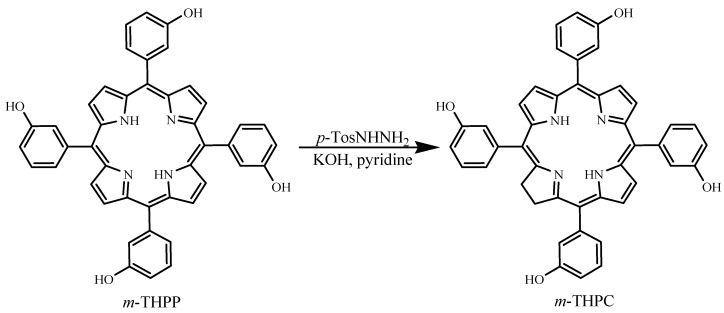
Formation of *meta*-tetra(hydroxyphenyl)chlorin (m-THPC) by tosylhydrazine reduction of *meta*-tetra(hydroxyphenyl)porphyrin (*m*-THPP).

*m*-THPC can be prepared by a reduction of one of the pyrrole rings in *m*-THPP, as shown in Figure 11 [2]. PDT treatment of neck and scalp cancer with *m*-THPC was approved in Europe, and the drug was used successfully for treating breast, prostate, and pancreatic cancers [48,50,51]. Light activation at 652 nm is very effective and only small doses of *m*-THPC are required during treatment. A weakness of *m*-THPC is high skin photosensitivity in some patients. 

SnET2, under the trademark Purlytin™, has been evaluated in Phase I/II trials for the treatment of metastatic breast adenocarcinoma, basal cell carcinoma, and Kaposi’s sarcoma [52]. This drug has also finished Phase III trials for the treatment of age-related macular degeneration but has not yet been approved by the FDA, due to a requirement of further efficacy and safety assessments [9]. Purlytin™ is activated at 664 nm and has deeper tissue penetration than Photofrin^®^. The drawback of the drug is a possibility of dark toxicity and skin photosensitivity.

NPe6 is another photosensitizer that can be irradiated at 664 nm for potential PDT treatment of fibrosarcoma, liver, brain, and oral cancer, and was approved in Japan in 2003 to treat lung cancer [31,53]. Similar to BPD-MA, NPe6 causes minimal skin photosensitivity, unlike Photofrin^®^.

#### 2.3.3. Pheophorbides

**Figure 12:**
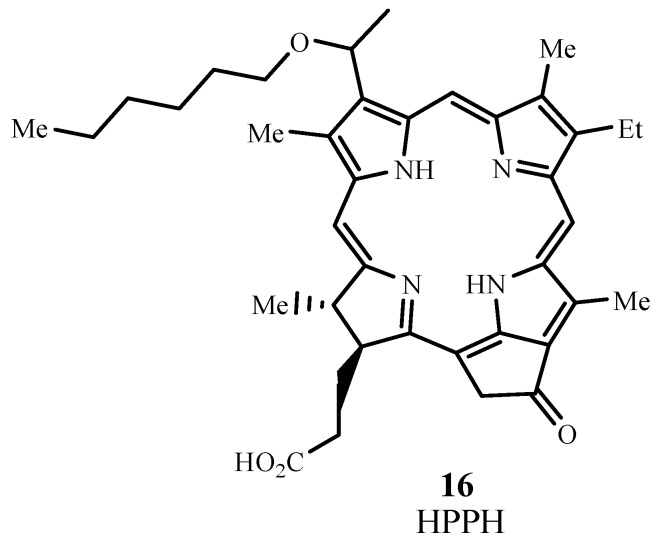
Molecular structure of 2-(1-hexyloxyethyl)-2-devinyl pyropheophorbide (HPPH).

Pheophorbides also have two extra hydrogens in one pyrrole unit and they can be derived from chlorophyll. HPPH is 2-(1-hexyloxyethyl)-2-devinyl pyropheophorbide (Photochlor^®^, **16**; Figure 12) and absorbs at 665 nm with ε_max_ ~ 47,000 M**^−1^** cm**^−1^** [54]. HPPH has been approved for use in clinical trials and has undergone Phase I/II trials for esophageal cancer (NCT00060268) [55] and Phase I trials involving basal cell skin cancer (NCT00017485) [56]. HPPH is currently in Phase II trials for lung cancer (NCT00528775) [57] and esophageal cancer at precancerous or early stage conditions (NCT00281736) [58], in Phase I trials for treating dysplasia, carcinoma of the oral cavity, carcinoma of the oropharynx (NCT01140178) [59], and head and neck cancer (NCT00670397) [60], and in phase I/II trials involving Barrett’s esophagus (NCT01236443) [61].

#### 2.3.4. Bacteriopheophorbides

**Figure 13:**
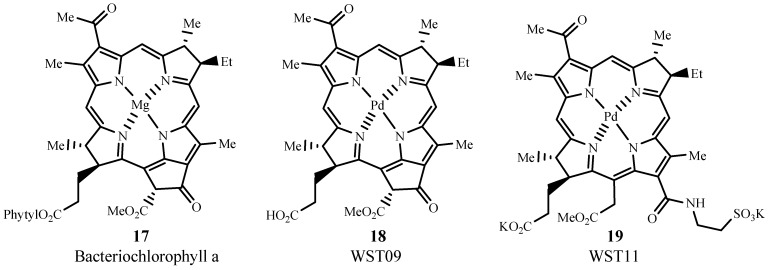
Examples of pheophorbide sensitizers for PDT use.

Bacteriopheophorbides are similar to bacteriochlorins but their structures have four more hydrogens than the corresponding porphyrins (cf. Figure 13). Generally, the λ_max_ of these sensitizers is red-shifted to 740–800 nm, with ε_max_ ~ 50,000 M**^−1^** cm**^−1^**. WST09 (padoporfin, Tookad^®^, **17**) is derived from bacteriochlorphyll a (**18**), which is isolated from bacteria. WST09 is activated at 763 nm and has low skin accumulation with quick drug excretion from the body [62]. This dye sensitizer has been evaluated for treating prostate cancer in Phase II clinical trials (NCT00308919) [63]. The advantages of deeper tissue penetration than Photofrin^®^ and minimum skin photosensitivity render this drug superior to other clinically used photosensitizers to date [52]. A water soluble derivative of WST09 is WST11 (Stakel^®^, **19**) and is manufactured by Steba Biotech [64]. Phase I/II (NCT00946881) [65] and Phase II (NCT00707356) [66] trials involving prostate cancer have been completed for WST11 in the US.

#### 2.3.5. Texaphyrins

**Figure 14:**
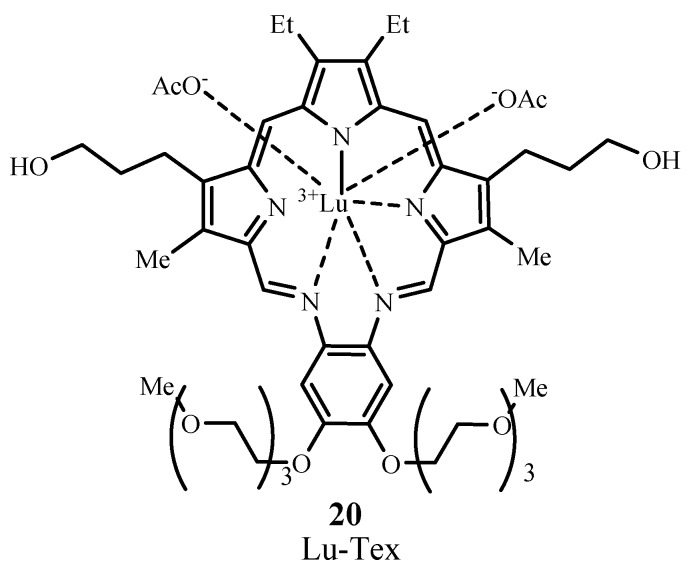
Molecular structure of a texaphyrin sensitizer.

Lu-Tex (motexafin lutetium, Lutrin^®^, **20**; Figure 14) is a texaphyrin, which is a porphyrinoid analog having a pentaaza core. This sensitizer is water soluble and absorbs light at 732 nm with ε_max_ ~ 42,000 M^−1^ cm^−1^. The drug Lutrin^®^ has been evaluated in Phase I trials for the treatment of prostate cancer, but requires further studies to confirm efficacy and to improve drug delivery [67]. Lutrin^®^ has undergone Phase I trials for treating cervical cancer (NCT00005808) [68], and has also entered Phase II trials for treatment of breast cancer and malignant melanoma [69]. Lu-Tex, under the trademark name Optrin^®^, is undergoing Phase II trials for the treatment of age-related macular degeneration, and Antrin^®^, also Lu-Tex, is undergoing clinical trials in photoangioplasty to treat peripheral arterial disease and coronary arterial disease [70]. 

#### 2.3.6. Phthalocyanines

**Figure 15:**
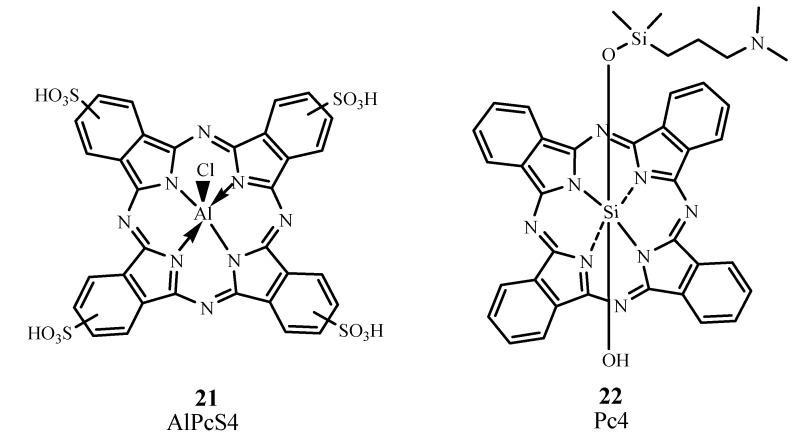
Examples of phthalocyanine PDT sensitizers.

Phthalocyanines (Pc) require metal complex formation to exhibit PDT properties because transition metals allow intersystem crossing to occur [71]. Their λ_max_ can be found at 670−700 nm, with ε_max_ ~ 200,000 M^−1^ cm^−1^. One specific Pc derivative is aluminum phthalocyanine tetrasulfonate AlPcS4, Photosens, **21; **Figure 15) which has λ_max_ at 676 nm. AlPcS4, as Photosens, has been used in Russia to treat stomach, skin, lip, oral, and breast cancer [9]. However, Photosens produces skin phototoxicity for several weeks.

Silicon phthalocyanine 4 (Pc4, **22**) is a phthalocyanine that absorbs at 675 nm and has completed Phase I trials for treating actinic keratosis, Bowen’s disease, skin cancer, and State I or II mycosis fungoides (NCT00103246) [72]. In preliminary studies involving cutaneous cancers such as recurrent breast cancer, a complete pharmacokinetic assessment and the maximum tolerated dose were not established [73]. 

A summary of the properties of clinical photosensitizers covered in this chapter is provided in Table 2 along with Φ_Δ_, where available [74,75,76]. 

**Table 2:** Properties of some photosensitizer dyes approved for PDT treatment and used in PDT-related clinical trials.

Compound	Trademark	λ_max_ (nm) ε_max_ (M^−1^ cm^−1^)	Φ_Δ_	Application
Porfimer sodium	Photofrin	632 (3000)	0.89	Canada (1993)—bladder cancer; USA (1995)—esophogeal cancer; USA (1998)—lung cancer; USA (2003)—Barrett’s esophagus; Japan—cervical cancer; Europe, Canada, Japan, USA, UK—endobroncheal cancer
5-Aminolevulinic acid (ALA)	Levulan	632 (5000)	0.56	USA (1999)—actinic keratosis
Methyl aminolevulinate (MAL)	Metvixia	–	–	USA (2004)—actinic keratosis
Hexaminolevulinate (HAL)	Cysview	–	–	USA (2010)—bladder cancer diagnosis
Benzoporphyrin derivative monoacid ring A (BPD-MA)	Visudine	689 (34,000)	0.84	USA (1999)—age-related macular degeneration
Meta-tetra(hydroxyphenyl)chlorin (*m*-THPC)	Foscan	652 (35,000)	0.87	Europe-neck and head cancer
Tin ethyl etiopurpurin	Purlytin	664 (30,000)	–	Clinical trials—breast adenocarcinoma, basal cell carcinoma, Kaposi's sarcoma, age-related macular degeneration
*N*-aspartyl chlorin e6 (NPe6)	Laserphyrin, Litx	664 (40,000)	0.77	Japan (2003)-lung cancer
2-(1-Hexyloxyethyl)-2-devinyl pyropheophorbide (HPPH)	Photochlor	665 (47,000)	–	Clinical trials—esophogeal cancer, basal cell carcinoma, lung cancer, Barrett’s esophagus
Palladium bacteriopheophorbide (WST09)	Tookad	763 (88,000)	0.50	Clinical trials—prostate cancer
WST11	Stakel	–	–	Clinical trials—prostate cancer
Motexafin lutetium (Lu-Tex)	Lutrin, Optrin, Antrin	732 (42,000)	–	Clinical trials—prostate cancer, age-related macular degeneration, breast cancer, cervical cancer, arterial disease
Aluminum phthalocyanine tetrasulfonate (AlPcS4)	Photosens	676 (200,000)	0.38	Russia (2001)—stomach, skin, lips, oral cavity, tongue, breast cancer
Silicon phthalocyanine (Pc4)	–	675 (200,000)	–	Clinical trials—actinic keratosis, Bowen’s disease, skin cancer, mycosis fungoides

### 2.4. Non-Porphyrin Photosensitizers

Although porphyrinoid structures comprise a majority of photosensitizers, several non-porphyrin chromogens exhibit photodynamic activity. These compounds include anthraquinones, phenothiazines, xanthenes, cyanines, and curcuminoids.

#### 2.4.1. Anthraquinones

**Figure 16:**
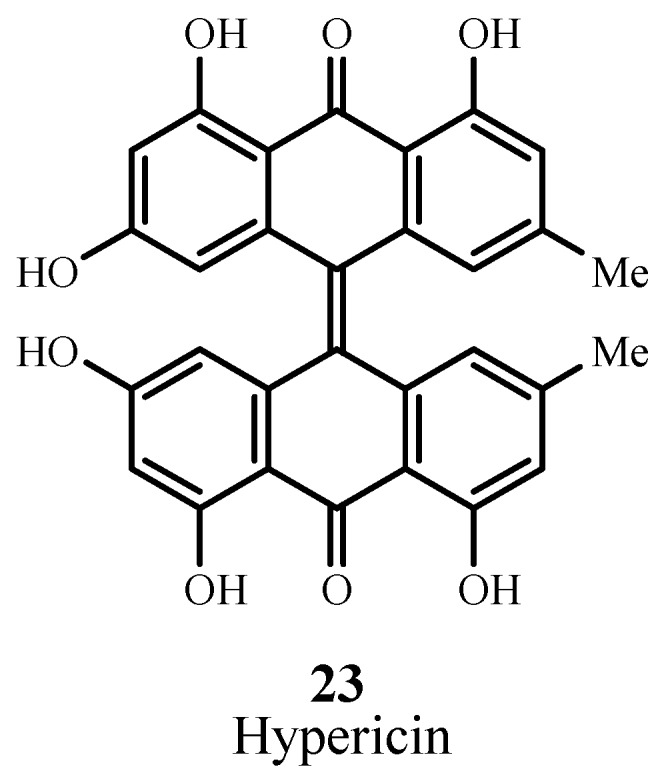
Example of an anthraquinone PDT sensitizer.

Hypericin (**23**; Figure 16) is a naturally occurring anthraquinone derivative extracted from St. John’s wort and is known for generating ROS that target cancer cells. Hypericin absorbs at 590 nm with ε_max_ ~ 44,000 M^−1^ cm^−1^. Clinical trials have been performed to treat squamous cell carcinoma and basal cell carcinoma [77] but the results are unsatisfactory to date. Studies aimed at optimization and enhancement of dosage, drug and light delivery, and preparation of tested area have been undertaken [78].

#### 2.4.2. Phenothiazines

**Figure 17:**
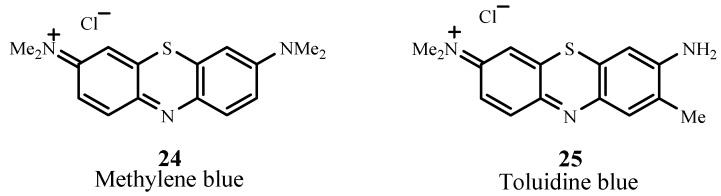
Examples of phenothiazine PDT sensitizers.

Methylene blue (**24**; Figure 17) belongs to the phenothiazinium family and absorbs at 666 nm with ε_max_ ~ 82,000 M^−1^ cm^−1^. This sensitizer targets melanoma cells and has positive PDT action against melanoma cell cultures [79]. Clinical PDT treatments using methylene blue include basal cell carcinoma and Kaposi’s sarcoma, *in vitro* testing of adenocarcinoma, bladder carcinoma, and HeLa cervical tumor cells [80]. Clinical trials involving chronic periodontitis have also been completed (NCT01535690) [81]. Another phenothiazinium dye is toluidine blue (25) which is undergoing Phase 2 clinical trials for treating chronic periodontitis (NCT01330082) [82]. Toluidine blue absorbs at 596 nm and 630 nm with ε_max(630 nm)_ ~ 51,000 M^−1^ cm^−1^ [83,84].

#### 2.4.3. Xanthenes

**Figure 18:**
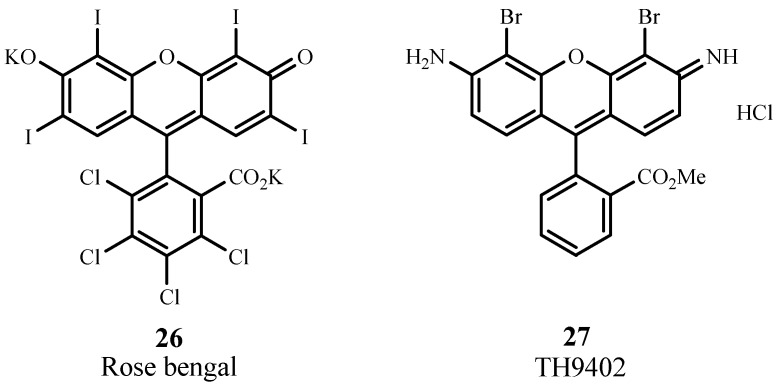
Examples of xanthene PDT sensitizers.

Rose Bengal (**26**; Figure 18) is a water soluble xanthene sensitizer that absorbs at 549 nm with ε_max_ ~ 100,000 M^−1^ cm^−1^. This sensitizer is an experimental agent for PDT treatment of breast carcinoma and metastatic melanoma [85]. 4,5-Dibromorhodamine methyl ester (TH 9409, 27) absorbs light at 514 nm with ε_max_ ~ 100,000 M^−1^ cm^−1^. The presence of halogen atoms increases the efficiency of intersystem crossing to the triplet state and yields singlet oxygen. This sensitizer has been evaluated for PDT treatment of graft-versus-host disease and it destroys lymphocytes via apoptosis [86,87]. It has also entered clinical trials involving allogeneic stem cell transplantation [9].

#### 2.4.4. Cyanines

**Figure 19:**
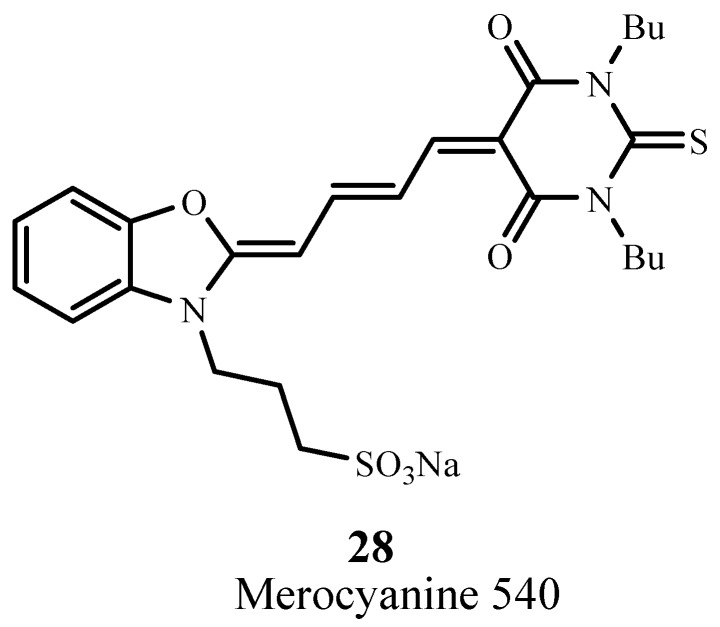
Example of a merocyanine PDT sensitizer.

Merocyanine 540 (**28**; Figure 19) absorbs at 556 nm with ε_max_ ~ 110,000 M^−1^ cm^−1^ and targets leukemia and lymphoma cells [88]. This cyanine sensitizer has been evaluated for PDT in preclinical and *in vitro* models for treatment of leukemia and neuroblastoma where it produced considerable cellular damage [89].

#### 2.4.5. Curcuminoids

**Figure 20:**
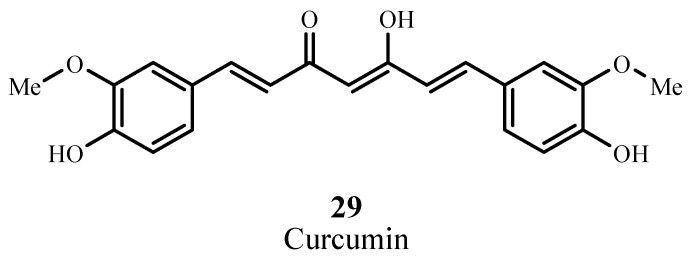
Example of a curcuminoid PDT sensitizer.

Curcumin (**29**; Figure 20) is a natural colorant isolated from rhizomas of *Curcuma longa L* and is a component of turmeric, a cooking spice [90]. Curcumin absorbs at 420 nm and has ε_max_ ~ 55,000 M**^−1^** cm**^−1^** [91], and has been used in a pilot study as a disinfectant in oral surgery via photodynamic action [92]. This natural dye has been proposed as an agent for destroying bacteria via PDT. 

The non-porphyrin sensitizers and their properties are listed in Table 3. None have received FDA approval for their application areas.

**Table 3:** Examples of non-porphyrin PDT candidates.

Compound	λ_max_ (nm)	ε_max_ (M^−1^ cm^−1^)	Application
Hypericin	590	44,000	squamous cell carcinoma, basal cell carcinoma
Methylene blue	666	82,000	melanoma, basal cell carcinoma, Kaposi’s sarcoma, chronic periodontitis
Toluidine blue	630	51,000	chronic periodontitis
Rose bengal	549	100,000	breast carcinoma, melanoma
TH9402	514	100,000	graft-versus-host disease
Merocyanine 540	556	110,000	leukemia, lymphoma
Curcumin	420	55,000	oral disinfectant

## 3. Conclusions

Once Photofrin^®^ was approved in Canada as a PDT sensitizer for treating bladder cancer, the development of photosensitizer dyes having improved PDT efficacy and low skin sensitivity became an important undertaking worldwide. However, only a few dye sensitizers, all porphyrinoid compounds, have been approved by regulatory authorities for use in PDT since the early days of Photofrin^®^. Although Photofrin^®^ is known to have the highest Φ_Δ_ and approval to treat many more cancer types than any other sensitizer, inconsistencies in its production, prolonged skin sensitivity after treatment completion, and low level of tissue penetration cause Photofrin^®^ to be far from ideal. Interestingly, WST09, due to its production reproducibility, absorption properties for deeper tissue penetration, efficacy in clinical trials, and minimal skin sensitivity has better overall properties. It has not yet been approved for PDT treatments. 

Currently, porphyrinoid sensitizers enjoy a few advantages over non-porphyrin sensitizers, including longer λ_max_ (630–760 nm) and research attention extending well into clinical trials. Non-porphyrin sensitizers have λ_max_ range ~420–670 nm with the majority of these chromogens having λ_max_ below 600 nm. Most of the non-porphyrins sensitizers have been and are still used in medicine, due to their antibacterial, antiviral, antimicrobial, and staining properties on biological tissues. There is a need for further research to modify these chromogens to extend their absorptions past 700 nm. Additionally, very little is known about the photophysical and pharmacokinetic properties of non-porphyrin sensitizers reported in this review. Additional preclinical studies need to be undertaken to determine optimal delivery methods, potency, irradiation source, and accumulation in and removal from post-treatment. 
